# Alkylated Benzodithienoquinolizinium Salts as Possible Non-Fullerene Organic N-Type Semiconductors: An Experimental and Theoretical Study

**DOI:** 10.3390/ma14216239

**Published:** 2021-10-20

**Authors:** Andrés Aracena, Marcos Caroli Rezende, Macarena García, Karina Muñoz-Becerra, Kerry Wrighton-Araneda, Cristian Valdebenito, Freddy Celis, Octavio Vásquez

**Affiliations:** 1 Instituto de Ciencias Naturales, Universidad de las Américas, Manuel Montt 948, Santiago 7500000, Chile; 2 Facultad de Química y Biología, Universidad de Santiago de Chile, Santiago 9160000, Chile; marcos.caroli@usach.cl; 3 Laboratorio de Procesos Fotónicos y Electroquímicos, Facultad de Ciencias Naturales y Exactas, Universidad de Playa Ancha, Valparaíso 2340000, Chile; macarena.garcia@upla.cl (M.G.); freddy.celis@upla.cl (F.C.); 4 Dirección de Investigación y Postgrado, Universidad de Aconcagua, Pedro de Villagra 2265, Santiago 7630000, Chile; karina.munoz@uac.cl; 5 Programa Institucional de Fomento a la Investigación, Desarrollo e Innovación, Universidad Tecnológica Metropolitana, Ignacio Valdivieso 2409, Santiago 8940577, Chile; Kerry.wrighton@utem.cl; 6 Centro Integrativo de Química y Biología Aplicada (CIBQA), Facultad de Ciencias de la Salud, Universidad Bernardo O’Higgins, Santiago 8320000, Chile; cristian.valdebenito@usach.cl; 7 Facultad de Ciencias Físicas y Matemáticas, Universidad de Chile, Santiago 8320000, Chile; svasqueza@ing.uchile.cl

**Keywords:** benzodithienoquinolizinium cations, photocyclization of N-aryl pyridinium salts, π-stacked assemblies, organic semiconductors

## Abstract

Three photobicyclized benzodithienoquinolizinium tetrafluoroborates (BPDTQBF4) were prepared and evaluated by UV–Vis and fluorescence spectral, electrochemical analysis, and by theoretical calculations as possible organic n-type semiconductors. Evaluation and comparison of their LUMO levels, HOMO-LUMO energy gaps as monomeric and π-stacked dimers with those of other materials, suggest their potential as organic n-type semiconductors. Calculations of their relative charge carrier mobilities confirmed this potential for one derivative with a long (C-14) alkyl chain appended to the polycyclic planar π-system.

## 1. Introduction

The development of new and more efficient organic semiconductors is an active area of research with a plethora of applications. They are normally classified as p-type (electron-donating or hole-conducting), n-type (electron-accepting or electron-conducting), and ambipolar (hole- and electron-conducting) semiconductors. The preparation and the study of electron-donating or p-type semiconductors have received considerable attention [1]. Compared to them, the development of their n-type counterparts has been left behind due in part to their greater atmospheric sensitivity and the rather limited number of molecular scaffolds employed for their synthesis. Research has been mainly concentrated on fullerene derivatives because of their high electron affinity and electron mobility [2,3,4,5,6]. Due to their ease of polymer intercalation and efficient electron transport, fullerene derivatives became an important class of electron acceptors for highly efficient organic solar cells, particularly when coupled with high-performance, electron-donating polymers [7]. Table 1 shows a comparison of some properties of fullerene derivatives and small molecules, when they are used as n-type semiconductors in organic solar cells.

Despite these advantages, however, recent studies have suggested that degradation of organic solar cells may be due to their high photo- and oxygen-sensitivity, and their tendency to form macroscopic aggregates [14,15,16]. Fullerene and its derivatives are weak absorbers of visible light [17,18]. This feature results in poor light-harvesting properties, thereby substantially reducing the potential of the photocurrent generation of organic solar cells in the UV–Visible range of the solar spectrum. These limitations and the poor synthetic flexibility shown by fullerene have led to efforts to develop other organic n-type semiconductors with a non-fullerene scaffold. Research in this field has concentrated on various oligomers [19,20] and polymeric materials [21,22], with few examples of small molecules.

A variety of oligomeric *N*-hydrogenated/*N*-methylated pyridinium salts have been used as cathode interfacial layers to increase the electron-mobility without modifying the work function of the electrode [23,24]. Oligothiophenes [25,26,27] have exhibited decreased LUMO energies, together with increased electron mobilities. Many of these properties can be anticipated theoretically, allowing for the identification of potentially interesting materials for the development of n-type organic semiconductors. An example of such computational studies is the theoretical calculation of HOMO-LUMO energy gaps of thiophene-based discotic systems, their charge-transfer rates, and relative charge-mobilities [28,29,30,31]. Theoretical structural and electronic properties of these kinds of compounds have been studied [32,33,34,35]. A series of ten 2-naphthyl and 2-anthrylbithiophene derivatives were synthesized and studied as possible n-type semiconductors [36]. Spectroscopic measurements and X-ray analyses were carried, together with theoretical calculations with DFT methods. Some experimental properties were in good agreement with the theoretical predictions. Theoretical and experimental studies with monomeric and dimeric DAE molecules have been carried out [37]. Theoretical calculations showed that the π extension of the dimer produced a decrease in the LUMO energy by comparison with the monomer. Low values for the internal reorganization energies (λ) were obtained for the dimer when compared with the monomer, leading to a higher semiconductor performance of the former. 

Polycyclic aromatic hydrocarbons (PAHs) have been proposed as semiconductors due to their ability to produce a discotic stacking, allowing an efficient charge-transport through their vertical intermolecular axis [38,39,40]. Polycyclic pyridinium salts are positively charged PAHs [41,42] and they constitute a class of electron-deficient planar π-system whose versatile synthesis can include other electron-donor or acceptor groups. Recently, pyridinium-based molecules have been used in dye-sensitized solar cells [43,44]. A pyridinium fragment, appended to a polyelectrolyte system, enabled efficient electron transport/collection, giving rise to a PCE value of 16.14% in an organic solar cell [45].

The above features of pyridinium- and thiophene-based extended π-systems and their possible formation of a discotic stacking have attracted our attention as a new class of electron-deficient systems with potential use in solar cells. To the best of our knowledge, photocyclized pyridinium salts have never been proposed as potential n-type organic semiconductors. Their synthesis by photocyclization of readily obtained *N*-arylpyridinium salts has been described for more than thirty years [46,47]. More recently, some of their derivatives such as 2-phenyl-benzo[8,9]quinolizino[4,5,6,7-fed]-phenanthridinylium tetrafluoroborate (PQPBF_4_) have been shown to form columnar π-stacked aggregates in the solid phase [48]. Although such aggregation in acetonitrile has been disputed [49], spectral evidence for its formation in water has been obtained in our laboratories [50]. Thus, planar electron-deficient π-systems like PQP^+^ or its dithienoquinolizium analog BPDTQ^+^ should be good candidates for promising n-type semiconductors. The incorporation of side-chains of variable size into PQP^+^ derivatives has led to materials with different discotic stackings in the solid state [51,52], suggesting their use in supramolecular assemblies on electronic surfaces [53,54].

In line with other theoretical studies [55] that not only shed light on their properties, but also help identify new interesting compounds by comparing their properties with those of known materials, we investigate in the present communication the properties of polycyclic pyridinium salts as candidates for the development of n-type organic semiconductors. Besides their electron-deficient character and their potential ability to form columnar aggregates, other structural features were included in their synthesis, which were above-mentioned as leading to better n-type organic semiconductors. They include the presence of dithieno substituents to the pyridinium core, and of alkyl side-chains of variable size, all of which should be expected to improve the charge-mobility in these systems. The effect of these structural modifications on a discotic arrangement of these molecules was assessed by calculations of their electronic behavior, injection, and charge-transport properties, HOMO and LUMO energies, and supported by experimental determination of their thermal stability and oxidation/reduction potentials through electrochemical measurements.

## 2. Materials and Methods

### 2.1. Synthesis of Polycyclic Heteroaromatic Salts

Melting points were measured with a capillary Microthermal apparatus and were not corrected. ^1^H NMR spectra were obtained with a Bruker Avance 400 MHz and the samples were prepared in acetone-d_6_. The UV–Vis spectra were recorded with a Cary 50 spectrometer. Fluorescence spectra were recorded with a Perkin Elmer LS55 spectrofluorimeter.

The precursor 2,6-bis(2-thienyl)-4-phenylpyrylium tetrafluoroborate (**2**) was prepared following a reported procedure by condensation of benzaldehyde and 1-phenyl-3-(2-thienyl)propenone (**1**) in the presence of the diethyl ether–boron trifluoride adduct [56].

The preparation of the *N*-(4-alkylphenyl)-2,6-bis(2-thienyl)-4-phenylpyridinium tetrafluoroborate (**3a**–**c**) followed a general procedure by refluxing for 12 h in ethanol (30 mL) equimolar amounts of the precursor (**2**) and 4-alkylaniline (0.37 mmol), filtering the resulting precipitate, washing with cold ethanol, and recrystallizing from ethanol to give the corresponding fluoroborates (**3a**–**c**). In this way, the following fluoroborate salts were prepared:

***N*-(4-Hexylphenyl)-4-phenyl-2,6-bis(2-thienyl)pyridinium tetrafluoroborate (3a)**, yield 70%, mp 126–129 °C. ^1^H NMR (400 MHz, Acetone-d_6_) δ 8.71 (s, 2H), 8.30 (d, J = 7.2 Hz, 2H), 7.87 (d, J = 4.9 Hz, 2H), 7.80–7.63 (m, 7H), 7.43 (d, J = 8.1 Hz, 2H), 7.17 (t, J = 4.3 Hz, 2H), 2.86–2.68 (m, 4H), 1.77–1.57 (m, 2H), 1.32 (s, 4H), 0.91 (t, J = 6.0 Hz, 3H).

***N*-(4-Decylphenyl)-4-phenyl-2,6-bis(2-thienyl)pyridinium tetrafluoroborate (3b)**, yield 73%, mp 123–125 °C. ^1^H NMR (400 MHz, Acetone-d_6_) δ 8.71 (s, 2H), 8.30 (d, J = 7.3 Hz, 2H), 7.87 (d, J = 5.0 Hz, 2H), 7.80–7.64 (m, 7H), 7.43 (d, J = 8.0 Hz, 2H), 7.17 (t, J = 4.3 Hz, 2H), 2.86–2.69 (m, 4H), 1.73–1.60 (m, 2H), 1.32 (s, 12H), 0.91 (t, J = 6.3 Hz, 3H).

***N*-(4-Tetradecylphenyl)-4-phenyl-2,6-bis(2-thienyl)pyridinium tetrafluoroborate (3c)**, yield 64%, 122–124 °C. ^1^H NMR (400 MHz, Acetone-d_6_) δ 8.71 (s, 2H), 8.30 (d, J = 7.3 Hz, 2H), 7.87 (d, J = 5.0 Hz, 2H), 7.78–7.63 (m, 7H), 7.43 (d, J = 8.1 Hz, 2H), 7.17 (t, J = 4.4 Hz, 2H), 2.84–2.64 (m, 4H), 1.72–1.60 (m, 2H), 1.32 (s, 20H), 0.89 (t, J = 6.4 Hz, 3H).

The cyclized 2-phenyl-7-alkyl benzo[ij]pyrido[2,1,6-de]dithieno[3,2-b:2′,3′-g]quinolizinium tetrafluoroborate salts (**4a**–**c**) were prepared following a general procedure. A solution of the precursor **3a**–**c** (0.5 mmol) in 50 mL of a 1:5 *v*/*v* mixture of ethanol:hexane was irradiated for 80 h in a quartz flask in a Rayonet NORPR-100 photochemical reactor with four 300 nm lamps. The precipitated quinolizinium tetrafluoroborate 4**a**–**c** was filtered and recrystallized in ethanol. In this way, the following tetrafluoroborate salts (**4a**–**c**) were prepared:

**7-Hexyl-2-phenylbenzo[ij]pyrido[2,1,6-de]dithieno[3,2-b:2′,3′-g]quinolizinium tetrafluoroborate (Hexyl BPDTQBF4) (4a)**, yield 33%, mp 275 °C (dec.). ^1^H NMR (400 MHz, Acetone-d6) δ 9.05 (s, 2H), 8.92 (s, 2H), 8.62 (d, J = 5.21 Hz, 2H), 8.58 (d, J = 5.26 Hz, 2H), 8.42–8.35 (m, 2H), 7.77 (d, J = 5.21 Hz, 3H), 2.86–2.68 (m, 4H), 1.77–1.57 (m, 2H), 1.32 (s, 4H), 0.91 (t, J = 6.0 Hz, 3H).

**7-Decyl-2-phenylbenzo[ij]pyrido[2,1,6-de]dithieno[3,2-b:2′,3′-g]quinolizinium tetrafluoroborate (Decyl BPDTQBF4) (4b)**, yield 38%, mp 240 °C (dec). ^1^H NMR (400 MHz, Acetone-d6) δ 9.07 (s, 2H), 8.94 (s, 2H), 8.67–8.55 (m, 4H), 8.39 (d, J = 6.36 Hz, 2H), 7.77 (d, J = 5.31 Hz, 3H), 2.86–2.69 (m, 4H), 1.73–1.60 (m, 2H), 1.32 (s, 12H), 0.91 (t, J = 6.3 Hz, 3H).

**7-Tetradecyl-2-phenylbenzo[ij]pyrido[2,1,6-de]dithieno[3,2-b:2′,3′-g]quinolizinium tetrafluoroborate (Tetradecyl BPDTQBF4) (4c)**, yield 44%, mp 225 °C (dec). 1H NMR (400 MHz, Acetone-d6) δ 9.07 (s, 2H), 8.95 (s, 2H), 8.62 (q, J = 5.40 Hz, 4H), 8.38 (d, J = 6.00 Hz, 2H), 7.77 (d, J = 5.31 Hz, 3H), 2.84–2.64 (m, 4H), 1.72–1.60 (m, 2H), 1.32 (s, 20H), 0.89 (t, J = 6.4 Hz, 3H).

### 2.2. Electrochemical Measurements

Cyclic voltammetry experiments were carried out in a PalmSens 4 potentiostat/galvanostat/impedance analyzer, using a one-compartment cell with a conventional three-electrode arrangement. As the working electrode, a glassy carbon electrode (CH Instruments, TX, USA) was used; as a counter electrode, we used a Pt wire (CH Instruments, TX, USA) and as a reference, we used a Ag/AgCl (3 M KCl) electrode. This last electrode was sealed and separated using a glass tube connected to the solution through a platinum bridge, working as a Lugging capillary. This arrangement avoids any moisture contamination to the working solution at the timescale of the CV experiments [57]. Tetrabutylammonium perchlorate (TBPA) 0.1 M was employed as the supporting electrolyte in 1 mM solutions of the tetrafluoroborate salts in DMF, with scan rates of 100 mV/s.

### 2.3. Thermogravimetric Measurements

The thermogravimetric analysis was performed using a NETZSCH TG 209 **F1** Libra thermoanalyzer at a constant heating rate of 3 K/min in a range of 30–500 °C. A constant purging gas flow of 20 mL/min nitrogen was applied with a protective gas flow of 8 mL/min nitrogen. Data analysis was carried out with Proteus Software (version 6.1).

### 2.4. Computational Details

The optimized structures of the monomeric BPDTQ cations and of their tetrafluoroborate salts, and the various conformations of their dimers (parallel and face to face) were calculated with Gaussian 09 [58] employing the hybrid DFT-B3LYP and M062X functionals, with the 6-31G (d) basis set. The electronic spectra of monomeric and dimeric molecules in methanol were obtained by TD-DFT methods with the 6-31G (d) basis set. Solvent effects were mimicked with the polarized continuum model (PCM). Intermolecular non-covalent interactions within the various conformations of the dicationic dimers were depicted with the NCIPLOT software (version 3.0) [59].

Intermolecular orbital overlap for holes and electrons were calculated using the Koopman approximation.

## 3. Results

### 3.1. Synthesis of the Polycyclic Heteroaromatic Salts ***4a**–**c***

**Scheme 1:**
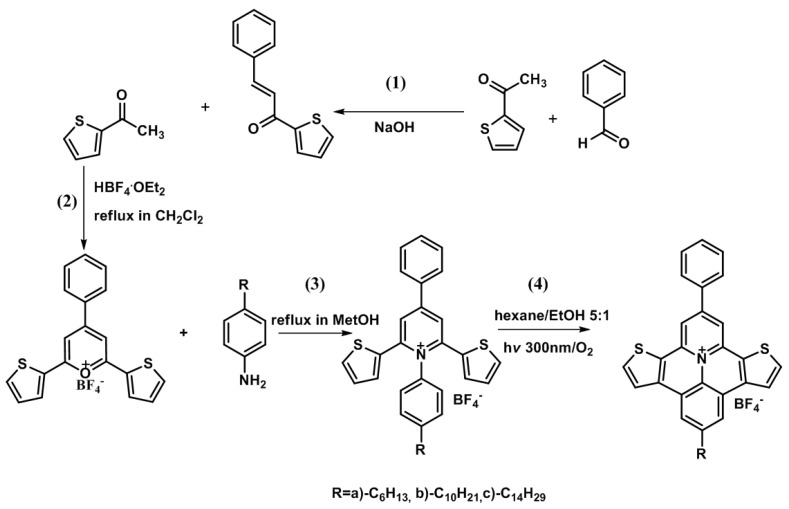
Synthetic route for the preparation of polycyclic heteroaromatic tetrafluoroborates **4a**–**c**.

The synthetic route to the heteroaromatic salts **4a**–**c** is shown in Scheme 1.

The basic condensation of 2-thienylethanone with benzaldehyde to form propenone (**1)**, followed by acid-catalyzed cyclization of this product with benzaldehyde to give the pyrylium tetrafluoroborate (**2**), are classical procedures reported previously [56]. Conversion of (**2)** into the corresponding *N*-aryl-2,6-bis(2-thienyl)-4-phenyl pyrydinium tetrafluoroborates **3a**–**c** by reaction with 4-alkylanilines also followed a standard procedure, described in the literature [46]. The photocatalytic bicyclization of *N*-aryl-2,4,6-trisubstituted pyridinium salts has been previously described [47] and the procedure was adapted to the preparation of tetrafluoroborate salts **4a**–**c** from the corresponding pyridinium salts **3a**–**c.**

### 3.2. Spectroscopic Characterization of Tetrasubtituted and Photocyclized Pyridinium Salts

**Figure 1:**
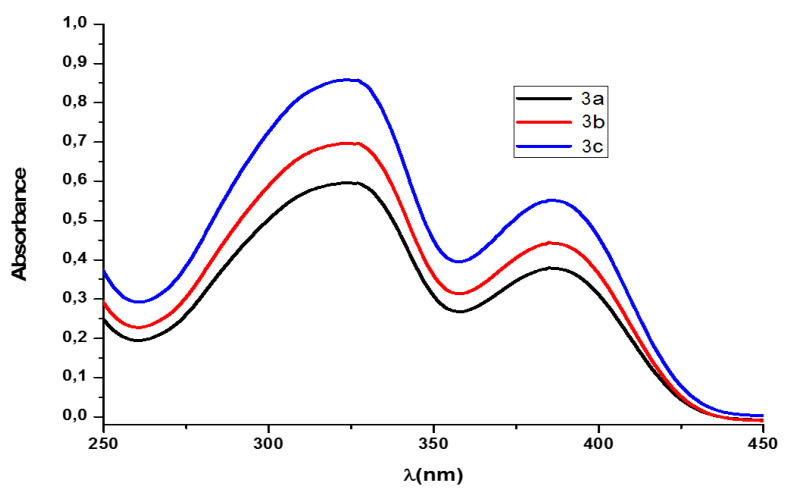
UV–Vis spectra of pyridinium tetrafluoroborates **3a**–**c** in chloroform (*ca.* 9 × 10^−4^ M).

The tetrasubtituted pyridinium salts **3a**–**c** showed two absorption maxima 327 and 312 nm, respectively, in chloroform (Figure 1). When the absorption spectra of these salts were measured in methanol as the solvent, two absorption maxima were also registered at 378 and 385 nm (7S). The spectra were not affected by the size of the alkyl substituent of the *N*-phenyl group, but there existed a hypsochromic shift of the maxima passing from the chloroform to methanol medium (−15 and −7 nm), probably because of the major stabilization of the ground state of the molecules with the polar protic solvent.

**Figure 2:**
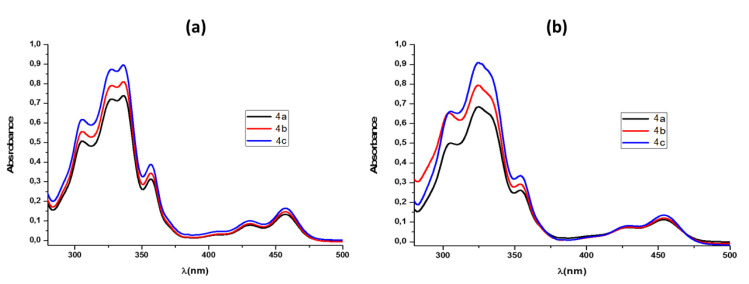
UV–Vis spectra of photocyclized salts **4a**–**c** (**a**) in chloroform (*ca.* 8 × 10^−4^ M) and (**b**) in methanol (*ca.* 8 × 10^−4^ M).

Upon cyclization, the extended heterocyclic π−system exhibited several new bands around 360, 430, and 470 nm, in addition to the major absorption around 330 nm, which consisted of at least two resolved bands (Figure 2).

The optical band gap (Eg) for compounds **4a**–**c** compounds could be estimated by the equation

(1) $$E_{g\;}=1241/\lambda_{\mathrm{onset}}$$

where λ_onset_ is the low-energy absorption edge obtained from the UV–Visible spectra in chloroform (Figure 2a) employing a geometric method [60]. From λ_onset_ values of 476, 475, and 477 nm for compounds **4a**, **4b**, and **4c**, respectively, E_g_ values of 2.61, 2.60, and 2.60 eV were obtained.

**Figure 3:**
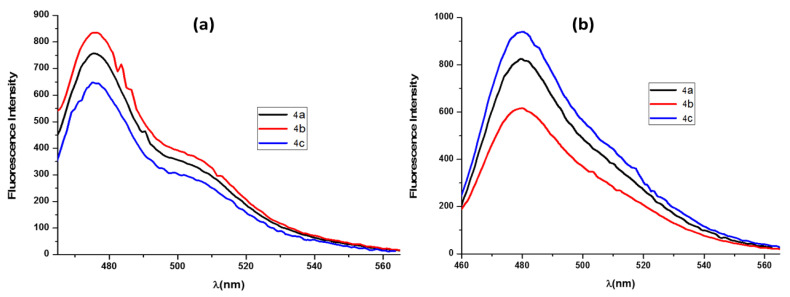
Fluorescence spectra of photocyclized salts **4a**–**c** (**a**) in chloroform (*ca.* 6 × 10^−6^ M), upon excitation at λ_max_^exc^ = 350 nm; (**b**) in methanol (*ca.* 8 × 10^−6^ M), upon excitation at λ_max_^exc^ = 350 nm.

Unlike salts **3**, the rigid polycyclic scaffold of **4a**–**c** prevented non-radiative decay processes of their excited species, leading to an emission band around 480 nm (Figure 3).

The comparison of the UV–Vis and fluorescence spectra of **4a**–**c** in chloroform and in methanol (Figure 2 and Figure 3) revealed some differences, but are not conclusive as to the possible formation of aggregates in the more hydrophilic methanolic medium. Discotic aggregation in methanol has been suggested for polycyclic systems analogous to **4** on the basis of dynamic light-scattering measurements [51]. Following this view of a postulated face-to-face aggregation in the solid phase of analogous highly planar hydrophobic structures [48], we cannot discard the formation of salt **4** aggregates in methanol. A more detailed investigation of this possibility, and of its consequences on the absorption spectra of these compounds and on their electron-deficiency was carried out with theoretical tools in Section 3.4 and Section 3.5.

### 3.3. Electrochemical Studies

Cyclic voltammetric studies of pyridinium salts **3** and of their cyclized derivatives **4** were carried out to gain information on the potentials of these compounds as electron-deficient materials, and on the effect of the bicyclization of **3** on these potentials.

**Figure 4:**
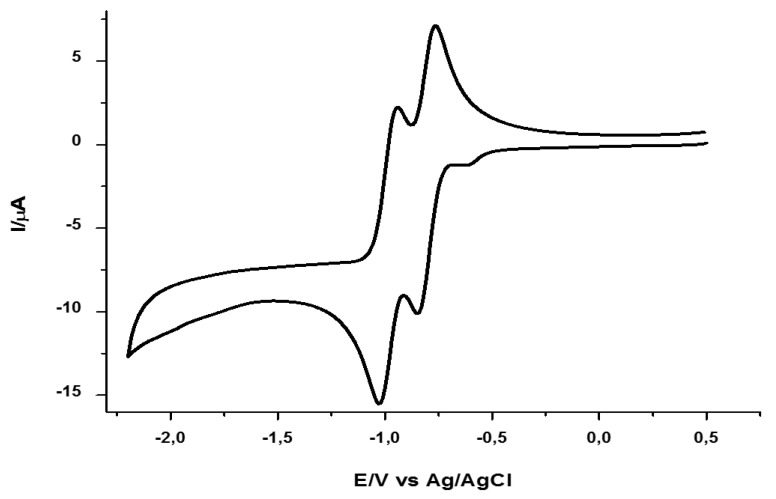
Cyclic voltammogram of pyridinium salt **3a** (1 mM) in DMF, with 0.1 M TBPA as a supporting electrolyte and a scan rate of 100 mV/s.

**Figure 5:**
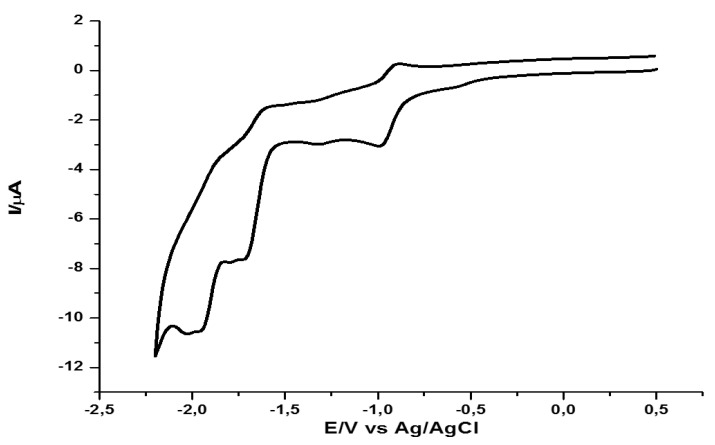
Cyclic voltammogram of the BPDTQ tetrafluoroborate **4a** (1 mM) in DMF, with 0.1 M TBPA as the supporting electrolyte and a scan rate of 10 V/s.

The nature of the alkyl substituent R in series **3a**–**c** and **4a**–**c**, did not alter the obtained voltammograms. Figure 4 presents the cyclic voltammogram of pyridinium tetrafluoroborate **3a**. The same experimental conditions were applied in obtaining the voltametric profile of the bicyclized derivative **4a** shown in Figure 5.

The potential of the first reduction process can be related to the electron affinity (EA) of the compounds. For this, it is necessary to relate the electrochemical potential to the vacuum level. Using Equation (1), it is possible to obtain the reduction potential (E_red_) relative to the vacuum level (E_vac_), the onset reduction potential (E^′^_red_), and the Ag/AgCl reference electrode (E_Ag/AgCl_) [61,62].

E_red_ = E^′^_red_ + E_Ag/AgCl_ ≈ (E^′^_red_ + E_vac_ + 4.4) eV (2)

which, by making E_vac_ = 0, reduces to

EA = −(E^′^_red_ + 4.4) eV (3)

The obtained values of E^′^_red_ for **4a**, **4b**, and **4c** were −0.80, −0.78, and −0.77 V.

Since no oxidation processes were observed, the HOMO energy level value can be calculated considering the optical band gap (E_g_) determined previously for each compound.

Table 2 lists the estimated values of the onset reduction potential (E^′^_red_), and of the HOMO and LUMO energies of BPDTQ tetrafluoroborates **4a**, **4b**, and **4c**.

For the sake of comparison, we list in Table 3 the HOMO and LUMO energy levels for a number of small molecules used as n-type organic semiconductors [9,10,11,12,13,63,64,65,66], most of them employed in organic solar cells.

Note that the HOMO energies of BPDTQ salts **4** fall within the range of corresponding values for n-type organic semiconductors, −6.68 (NDINI) < −6.37 (NDITz1) < −6.33 (IDTO-5Br) < −6.23 (**4c**) < −6.22 (**4b**) < −6.21 (PDI) < −6.20 (**4a**) < −6.15 (oo-PDI) < −6.10 (C8-NDTI). The same takes place with the LUMO energies, with values for the BPDTQ salts close to –3.60 eV (Table 2), which fall within the range of −3.49 to −4.20 eV, observed for the organic molecules in Table 3.

In order to produce a good ohmic contact between an n-type semiconductor and a cathode, and a favorable electron injection into the former, the difference between its LUMO and the cathode work function must be ideally smaller than 0.3 eV [67]. The estimated LUMO energies of the tetrafluoroborate salts **4a**–**c** (≈−3.6 eV), though slightly less negative than those of the other n-type organic semiconductors of Table 3, fulfils this requirement for commonly used electrodes such as Mg, with a work function of −3.66 eV.

### 3.4. Thermogravimetric Measurements

**Figure 6:**
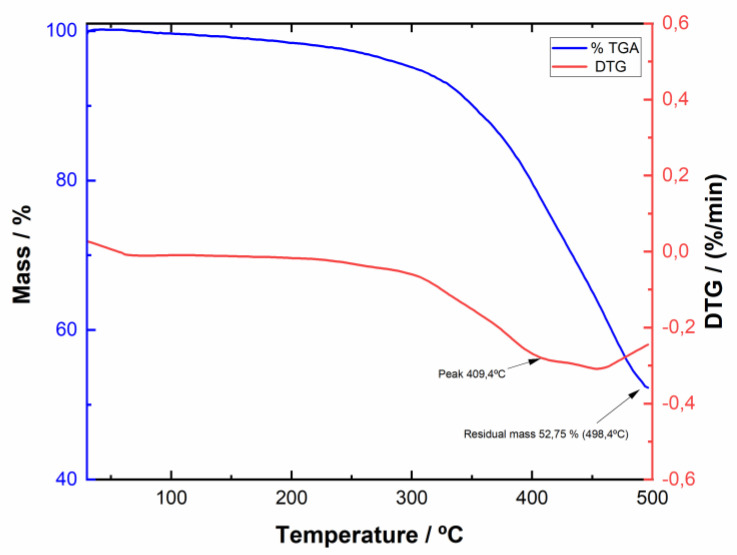
Thermogravimetric analysis of BPDTQ tetrafluoroborate **4c**.

The stability of BPDTQ tetrafluoroborates **4a**–**c** was next evaluated by thermogravimetric analysis. Figure 6 reproduces the TGA of compound **4c**, showing the loss in mass of the salt with the increased temperature. As can be seen, the BPDTQ salts were thermally very stable, only starting to decompose and lose mass at temperatures around 350 °C, thus ensuring their use in solar cells.

### 3.5. Theoretical Studies

In the previous sections, the potential of the photocyclized BPDTQ salts as promising organic n-type semiconductors was established by experimental estimates of their HOMO and LUMO energies, which fell within the range of frontier–orbital energies of other organic n-type semiconductors and led to an adequate ohmic contact between them and a metal cathode.

In order to gain a deeper insight into their behavior, we carried out theoretical calculations with three main purposes: (1) to have a clearer picture of the frontier orbitals and of the HOMO → LUMO transition of the isolated cations BPDTQ^+^; (2) to investigate the effect of π-stacking of these extended planar structures on their properties as n-type electron-accepting species; and (3) to have an insight into the electron-injection process from the BPTDQ^+^ cation into a metallic surface and to evaluate charge mobility in these species.

#### 3.5.1. Monomeric BPDTQ^+^ Cations

**Figure 7:**
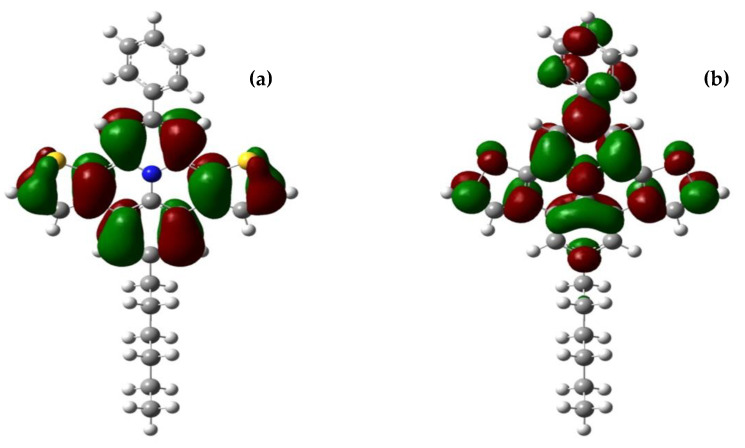
HOMO (**a**) and LUMO (**b**) densities of the **4a** cation in methanol, calculated using the B3LYP/6-31G(d) method and the PCM option.

Frontier–orbital calculations of the optimized structures of BPDTQ^+^ cations of **4a**–**c** were carried out with the hybrid DFT-B3LYP and M062X functionals, employing the 6-31G (d) basis set. Transition energies were obtained with TD-DFT calculations. Solvent effects were simulated with the PCM approach. Figure 7 reproduces the HOMO and LUMO of the **4a** cation. More detailed results from the frontier molecular orbital analyses are given in the Supplementary Materials.

The HOMO–LUMO transition involves a charge-transfer process from the benzo- and diethieno-rings to the quinolizinium and the 2-phenyl substituent.

Table 4 lists some calculated values for the **4a**–**c** cations, which can be compared to the experimentally-derived values of Table 4 and Table 5. In general, the nature of the appended alkyl chain did not affect the calculated values, so only the results for the 7-tetradecyl BPDTQ^+^ cation are given in Table 4.

A comparison of these values with those of Table 4 and Table 5 revealed a significant departure of theory from the experimentally-derived values. Calculations with the B3LYP/6-31g(d) method led to better results than with the DFT/M062X method, but still only tolerably reproduced the experimental values. LUMO levels in particular, were significantly lower than the experimental value of ≈−3.6 eV. As pointed out at the end of Section 3.3, caution is required when employing a HOMO–LUMO terminology based on different methods of calculation [68]. Calculated values of HOMO and LUMO energy levels may therefore differ from those estimated by optical or electrochemical measurements. By adopting a different method of calculation, with the assumption that the LUMO energy E_LUMO_ = −EA [69], the calculated LUMO energy of cation **4c** draws closer to the electrochemically-derived experimental value of −3.6 eV.

#### 3.5.2. π-Stacking in Dimeric BPDTQ^+^ Cations

Although the spectra of Figure 2 and Figure 3 did not offer sufficient evidence for the formation of aggregates in more polar methanol, reports of π-stacking in dimeric PQP^+^ [48,51] and in BPDTQ^+^ cations [50] led us to investigate the possible structures and electronic properties of the dimers of the **4a**–**c** cations.

**Figure 8:**
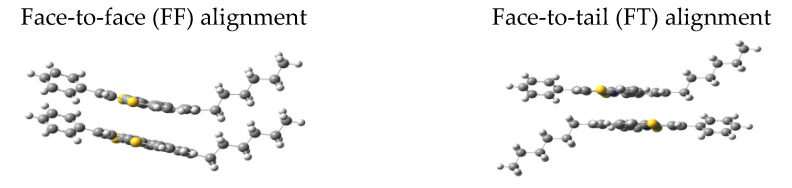
Face-to-face (FF) and face-to-tail (FT) alignments of dimers from cations of BPTDQ salt **4a**.

In the absence of data from crystal packing, we considered two forms of parallel stacking, one in which the two delocalized ring systems were aligned in the same direction (a face-to-face (FF) alignment) and another where they were aligned in opposite directions (face-to-tail (FT) alignment). The two conformations are reproduced in Figure 8 for the hexyl derivative **4a**.

Table 5 lists some calculated properties of these dimers: the distance between their π-stacked planes, their dipole moments, the interaction energies between the two monomers, and their longest-wavelength λ_max_ values.

**Figure 9:**
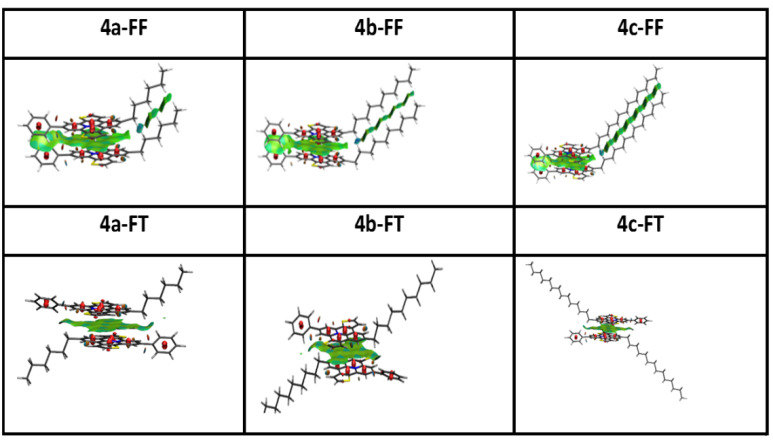
Face-to-face (FF) and face-to-tail (FT) π-stacked dimers of cations from 4**a**–**c**, with non-covalent interactions obtained from NCI analyses.

A non-covalent interaction (NCI) analysis was performed for all dimers of Table 5, and the results are summarized in Figure 9. More detailed results from the NCI analyses are given in the Supplementary Materials.

As can be seen, interactions are governed by π − π charge repulsions between the two cationic monomers, shown in red, which are compensated by a large, green iso-surface of attractive van-der-Waals interactions between the two polycyclic systems [70].

Interestingly, face-to-face dimers present an additional non-covalent interaction between the parallel alkyl chains, giving rise to an additional attraction between the two monomers. Such quasi-covalent interactions between parallel chains have been described previously [71]. This “fastener effect” between alkyl chains in solid samples should be responsible for an increased conductivity and electron mobility in their resulting films. More detailed NCI analyses are shown the in Supplementary Materials (Figure S10).

The dipole moments listed in Table 5 point to significant differences between the FF and the FT conformations, giving rise to correspondingly distinct interaction energies. Relatively non-polar FT dimers should be favored in non-polar media, whereas an FF alignment should predominate in polar solvents, giving rise to J-aggregates. In the latter media, the non-covalent attraction between the alkyl chains should provide an additional stabilization to these conformers.

A noteworthy result from Table 5 is the bathochromic shift of the longest-wavelength λ_max_ value of the dimers when compared with the corresponding values in Table 4 for the monomeric cations. Though small (<10 nm), using the B3LYP functional, this red shift points to a HOMO–LUMO gap for the discotic π-stacked aggregates of compounds **4a**–**c** smaller than the ones listed in Table 4 for the monomeric cations. Following previous suggestions of the formation of columnar aggregates in the solid phase [48] for analogous systems, leading to materials with different discotic stackings [51,52], our calculations point to a similar possibility for polycyclic systems **4a**–**c**, suggesting their use as n-type semiconductors in supramolecular assemblies.

#### 3.5.3. Charge Mobility in Dimeric BPDTQ^+^ Cations

Charge mobility is an important factor to be considered in organic semiconductors. Transport properties are commonly discussed with the aid of Marcus theory [55], though other approaches are also found in the literature [72,73,74,75,76,77,78].

Having assumed the two parallel alignments for our cationic dimers, we estimated the charge transport in these discotic systems employing the Marcus theory [55,79,80] and Einstein’s equation for a one-dimensional diffusion process [81,82].

**Scheme 2:**
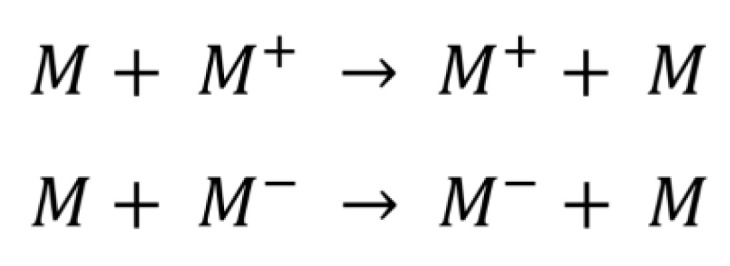
Hole/electron exchange between two molecules M in a hopping process.

According to the latter, the diffusion μ of a charge or a hole between two parallel surfaces separated by a distance l is given by Equation (4)

(4) $$\mu =\frac{\mathrm{eD}}{k_{B}T}=\frac{{\mathrm{el}}^{2}}{k_{B}T}{\;k}_{\mathrm{ET}}$$

where e is the electron charge; k_B_ is the Boltzmann’s constant; and k_ET_ is the charge-transfer rate constant between the two contiguous molecules. The k_ET_ value can be obtained from Equation (5), derived from Marcus theory

(5) $$k_{\mathrm{ET}}=\frac{4\pi^{2}}{h}\;\frac{1}{\sqrt{4{\pi \lambda k}_{B}T}}{\;t}^{2}\;\exp\;\left[\frac{-\lambda }{4k_{B}T}\right]\;$$

where t is the electronic coupling between the two contiguous molecules and λ is the reorganization energy of the system. The first term t depends on the distance between the parallel monomers and can be calculated using different methodologies [83]. In the present work, we employed Koopman’s methodology using the HOMO and LUMO energies of the interacting monomers [84,85,86], and the hopping process where a dimeric system of two molecules M exchange hole/electrons through a hole/electron-transfer reaction, as shown below in Scheme 2:

The reorganization energy λ of Equation (5) comprises an inner and an outer contribution. The first arises from rapid changes in the molecular geometries of the donor (D) and acceptor (A) species, and the latter from nuclear polarization/relaxation of the surrounding medium upon an electron transfer. In our case, this outer contribution can be neglected because our system is a static crystal pack [87]. Thus, the reorganization energy λ (inner) can be obtained from Equations (6), (7), and (10) for holes, and Equations (8)–(10) for electrons, where E(M) is the total energy of species M and the superscripts A and D refer to the electron-accepting and -donor molecules.

λ^A^ = E(M^+^) − E(M) (6)

λ^D^ = E(M) − E(M^+^) (7)

λ^A^ = E(M^−^) − E(M) (8)

λ^D^ = E(M) − E(M^−^) (9)

λ = λ^A^ + λ^D^ (10)

Table 6 lists the obtained values for parameters t and λ, μ, and k_ET_, for the mobility of electrons and holes in all cationic dimers from the BPDTQ^+^ salts.

An inspection of Table 6 revealed differences among the three dimers of **4a**–**c** and between each pair of FF/FT conformations. These variations point to possible differences in the behavior of these π-stacked dimers as semiconductors.

With the exception of **4c** dimers, the relative hole (μ**_h_**) and electron (μ**_e_**) mobilities of all dimeric species are of the same order of magnitude, with μ**_h_** < μ**_e_** for the FF conformations, and μ**_h_** >> μ**_e_** for the FT conformations. This amounts to predicting an ambipolar, or even a p-character for semiconductors obtained from these dimeric molecules. Dimers **4c** present a distinct behavior. For both conformers FF and FT, the relative hole mobility μ**_h_** is much smaller than the electron mobility μ**_e_**, anticipating for these dimeric species an n-type semiconductor behavior. Such low hole mobilities are a result of internal hole reorganization energies λ**_h_** almost four times greater than those of electrons λ**_e_**. The fact that dimers **4c**, with the longest C_14_ chain, show a strong n-type character, at variance with analogs **4a** and **4b** suggests that the size of the 7-substituent alkyl chain plays an important role in the charge-transport and mobility of these dimers. This echoes previous reports of changes in the hopping mobility of discotic alkylated hexabenzocoronenes with the increased size of the alkyl substituents [88]. Thus, trapping and recombination in these discotic materials are retarded by the increased size of the alkyl chains, a mechanism that may also operate for materials derived from **4c**.

## 4. Conclusions

Photobicyclization of *N*-aryl-2,6-dithienyl-4-phenyl pyridinium salts led to the formation of benzodithienoquinolizinium tetrafluoroborates (BPDTQBF_4_) that were investigated by spectral, electrochemical, and theoretical tools as possible n-type semiconductors for solar cells.

Their UV–Vis spectra and their voltamograms in DMF allowed for the estimation of their LUMO levels and HOMO–LUMO energy gaps, which had values compatible with those of other n-type semiconductors. Their UV–Vis spectra in chloroform and in methanol as well as their fluorescence in the same solvents did not show any conclusive evidence of aggregation, as might be surmised from reports of analogous systems in the literature. Nevertheless, theoretical calculations of the monomeric cations **4a**–**c** and of their π-stacked dimers in two different conformations (face-to-face, FF, and face-to-tail, FT) indicated that aggregation could lead to smaller HOMO–LUMO gaps, with a beneficial impact on the behavior of these materials as n-type semiconductors. In order to substantiate this expectation and obtain a deeper view on the many aspects that govern this complex behavior, preliminary calculations on their relative charge mobilities were performed. Estimates of relative charge mobilities for the two conformations of the cationic dimers from **4a**–**c**, based on Einstein’s diffusion equation and Marcus’ theory, led to inconclusive results, with the characterization of dimers from cation **4c**, with a long (C_14_) alkyl chain appended to the polycyclic core as a probable, potentially interesting n-type semiconductor.

In conclusion, in the present communication, the preparation of a new family of alkylated benzodithienoquinolizinium salts was described. Their electronic properties, estimated from spectroscopic and electrochemical measurements in solution, coupled with theoretical calculations in the gas-phase of their monomers and dimeric species, suggest their potential use as organic n-type semiconductors. In order to confirm their potentiality, measurements in the solid phase, and with thin films are necessary, aside from calculations based on their crystal structures. We will address these necessary issues in a future work, with the most promising compound of the series.

## Figures and Tables

**Table 1:** LUMO energy levels (obtained by electrochemical measurements), electron mobility values, donor/acceptor ratio and power conversion efficiency (PCE%) of fullerene derivatives and some organic small molecules used as n-type semiconductors.

Acceptor.	LUMO Energy (eV)	μ_e_ (cm^2^·V^−1^·s^−1^)	D/A Ratio	PCE (%)
PC_61_BM [8]	−3.70	2.39 × 10^−4^	1:0.7	3.80
NC_61_BM [8]	−3.68	2.27 × 10^−4^	1:0.7	4.09
AC_61_BM [8]	−3.75	1.75 × 10^−4^	1:0.7	1.14
PyC61BM [8]	−3.72	2.13 × 10^−4^	1:0.7	1.95
oo-PDI [9]	−3.90	-	1:1	8.12
PDI-3 [10]	−3.63	-	1:2	0.96
PDI-1 [10]	−3.67	-	1:2	0.13
NDITz [11]	−3.99	0.15	-	-
NDINI [12]	−4.20	1.75	-	-
C8-NDTI [13]	−4.00	0.05	-	-

**Table 2:** Onset reduction potential (E^′^_red_), LUMO energies, and HOMO data obtained.

Compound	E^′^red (V) vs. Ag/AgCl	LUMO (eV)	HOMO (eV)
**4a**	−0.80	−3.60	−6.20
**4b**	−0.78	−3.62	−6.22
**4c**	−0.77	−3.63	−6.23

**Table 3:** HOMO and LUMO energy values of various n-type organic semiconductors estimated under similar conditions [9,10,11,12,13,63,64,65,66].

Cpd.	LUMO Energy (eV)	HOMO Energy (eV)
T(m-BODIPY)_2_ [63]	−3.49	−5.53
Cz-BAR [65]	−3.51	−5.63
Flu-BAR [65]	−3.53	−5.81
PDI-3 [10]	−3.63	−5.75
PDI-1 [10]	−3.67	−5.79
PCBM [66]	−3.83	−5.55
oo-PDI [9]	−3.90	−6.15
PDI [9]	−3.92	−6.21
Fullerene [66]	−3.92	−5.71
NDITz1 [11]	−3.99	−6.37
C8-NDTI [13]	−4.00	−6.10
bo-PDI [9]	−4.02	−6.23
IDTO-5Br [64]	−4.12	−6.33
NDINI [12]	−4.20	−6.68

**Table 4:** Calculated values of frontier–orbital energies and corresponding absorption of the longest-wavelength band of the 7-tetradecyl BPDTQ^+^ cation in methanol, together with its ionization potential IP and electron affinity EA.

DFT Functional	HOMO Energy (eV)	LUMO Energy (eV)	HOMO-LUMO Energy Gap (eV)	λ_max_ of Longest-Wavelength Band (nm)	Ionization Potential IP (eV)	Electron Affinity EA (eV)
B3LYP	−8.84	−5.33	3.51	426	9.76	4.11
M062X	−10.04	−4.61	5.43	381	-	-

**Table 5:** Calculated properties of π-stacked dimers of **4a**–**c** cations.

Dimers ^a^	Distance between π-Stacked Planes (Å) ^b^	Dipole Moment (D) ^b^	Interaction Energy (kcal.mol^−1^) ^b^	Longest-Wavelength λ_max_ Value (nm) ^c^
**4a-FF**	3.29	7.49	14.38	430
**4a-FT**	3.31	0.11	15.51	432
**4b-FF**	3.24	20.90	11.74	430
**4b-FT**	3.32	2.24	15.00	433
**4c-FF**	3.24	36.41	8.38	430
**4c-FT**	3.33	1.74	14.76	433

^a^ Notations. **4a-FF**: cation from **4a** with a face-to-face alignment; **4a-FT**: cation from **4a** with a face-to-tail alignment; ^b^ in the gas-phase; ^c^ in methanol.

**Table 6:** Calculated values of the electronic coupling t, the reorganization energy λ, the charge-transfer rate constant k_ET_, and the relative mobility μ for all cationic dimers from **4a**–**c**.

Dimer	Electrons ^a^				Holes ^a^			
	10^2^ t_e_ (eV)	10 λ_e_ (eV)	10^−12^ k_ET_^e^ (s^−1^)	μ_e_ (cm^2^.V^−1^·s^−1^)	10^2^ t_h_ (eV)	10 λ_h_ (eV)	10^−12^ k_ET_^h^ (s^−1^)	μ_h_ (cm^2^·V^−1^·s^−1^)
**4a-FF**	3.36	1.9	7.10	0.30	2.14	1.95	2.69	0.115
**4a-FT**	3.80	1.9	9.05	0.38	16.4	1.96	158	6.65
**4b-FF**	5.17	2.0	14.7	0.63	2.56	2.0	3.46	0.148
**4b-FT**	4.87	2.0	13.0	0.53	9.28	2.0	45.5	1.86
**4c-FF**	2.67	1.95	4.19	0.18	10.20	7.9	0.089	3.84 × 10^−3^
**4c-FT**	4.25	1.95	10.6	0.43	0.41	7.9	1.49 × 10^6^	6.1 × 10^−8^

^a^ Subscripts e and h refer to electrons and holes, respectively.

## Data Availability

Not applicable.

## Supplementary Materials

The following are available online at https://www.mdpi.com/article/10.3390/ma14216239/s1, Figure S1: NMR spectrum of compound **3a** in acetone-d_6_. Figure S2: NMR spectrum of compound **3b** in acetone-d_6_. Figure S3: NMR spectrum of compound **3c** in acetone-d_6_. Figure S4: NMR spectrum of compound **4a** in acetone-d_6_. Figure S5: NMR spectrum of compound **4b** in acetone-d_6_. Figure S6: NMR spectrum of compound **4c** in acetone-d_6_. Figure S7: Absorption spectra of pyridinium salts **3a**, **3b**, and **3c** in methanol (*ca.* 9 × 10^−4^ M). Figure S8: Cyclic voltammograms obtained for tetrasubtituted pyridinium salts **3b** and **3c** in DMF (c = 1mM), with TBPA 0.1 M as the supporting electrode and a scan rate of 100 mV/s. Figure S9: Cyclic voltammograms obtained for BPDTQ salts **4b** and **4c** in DMF (c = 1 mM) with TBPA 0.1 M as the supporting electrode and a scan rate of 100 mV/s. Figure S10: NCI 3D representation of isosurfaces and analysis of the *s*((λ_2_)ρ) graph for the face-to-face (FF) and face-to-tail (FT) dimers. The color-scale code for the 3D representations is shown. Figure S11: Surfaces of HOMO (a) and LUMO (b) of the FT dimer of **4a^+^**. Figure S12: Surfaces of HOMO (a) and LUMO (b) of the FF dimer of **4a^+^**. Figure S13: Surfaces of HOMO (a) and LUMO (b) of **4b^+^**. Figure S14: Surfaces of HOMO (a) and LUMO (b) of the FT dimer of **4b^+^**. Figure S15: Surfaces of HOMO (a) and LUMO (b) of the FF dimer of **4b^+^**. Figure S16: Surfaces of HOMO (a) and LUMO (b) of **4c^+^**. Figure S17: Surfaces of HOMO (a) and LUMO (b) of the FT dimer of **4c^+^**. Figure S18: Surfaces of HOMO (a) and LUMO (b) of the FF dimer of **4c^+^**.
