# Supplementary material for: Alkylated Benzodithienoquinolizinium Salts as Possible Non-Fullerene Organic N-Type Semiconductors: An Experimental and Theoretical Study
Source: Materials (Basel). 2021 Oct 20;14(21):6239. doi: 10.3390/ma14216239 (PMC8584425; doi:10.3390/ma14216239)
Supplement: Supplementary file 1 [file materials-14-06239-s001.zip › materials-1391312-supplementary.pdf]

materials MDPI

Electronic Supplementary Material (ESI)

Supplementary information Alkylated benzodithienoquinolizinium salts as possible non- fullerene organic n-type semiconductors: an experimental and theoretical study.

Andrés Aracena*, Marcos Caroli Rezende, Macarena García, Karina Muñoz-Becerra, Kerry Wrighton-Araneda, Cristian Valdebenito, Freddy Celis and Octavio Vásquez.

1. H-NMR spectra of pyridinium salts 3a-c and BPDTQ salts 4a-c

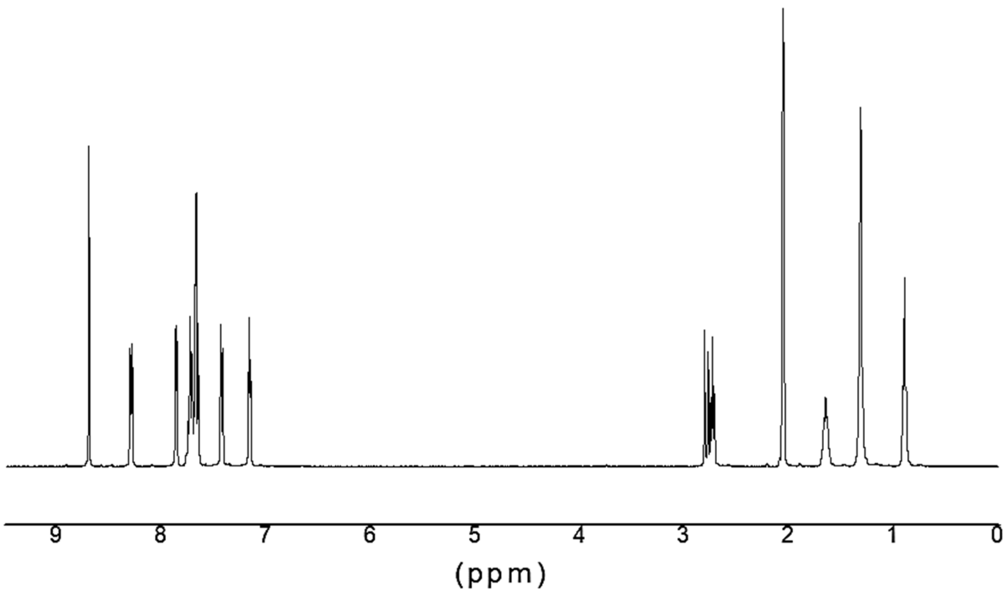

Figure S1. NMR spectrum of compound **3a** in acetone-d_6.

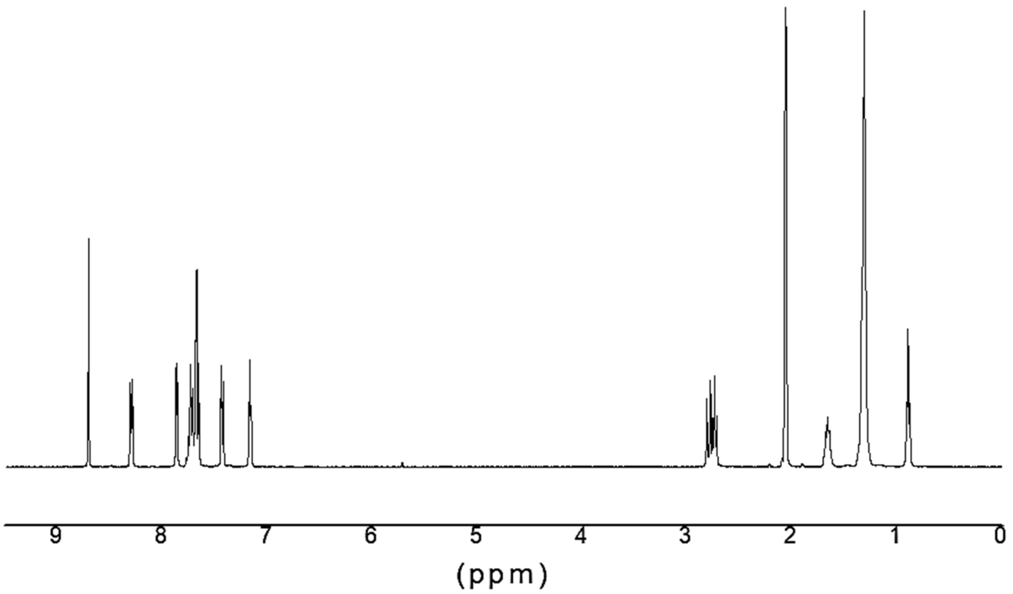

Figure S2. NMR spectrum of compound **3b** in acetone-d6.

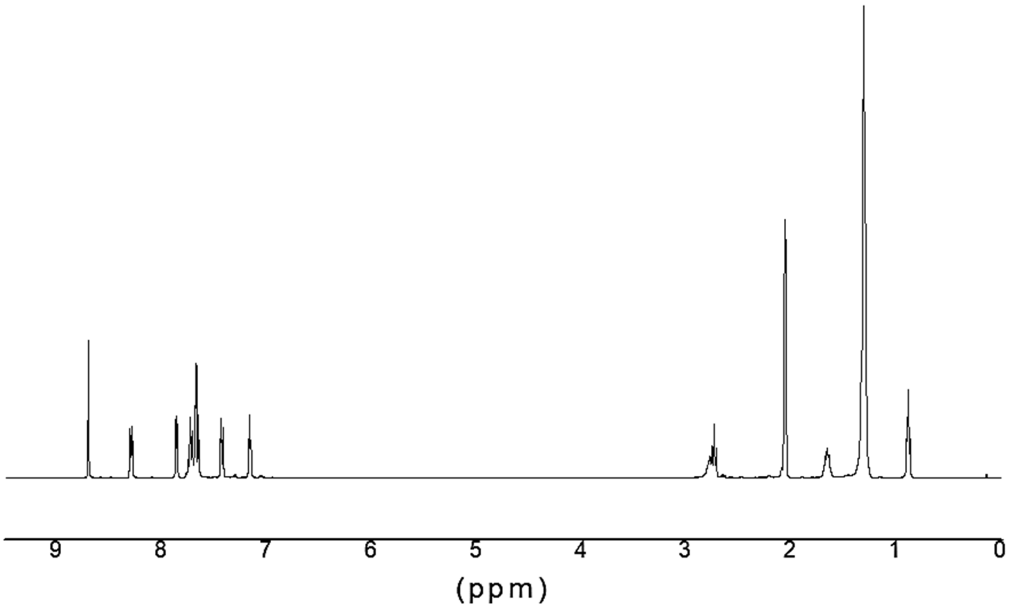

Figure S3. NMR spectrum of compound **3c** in acetone-d6.

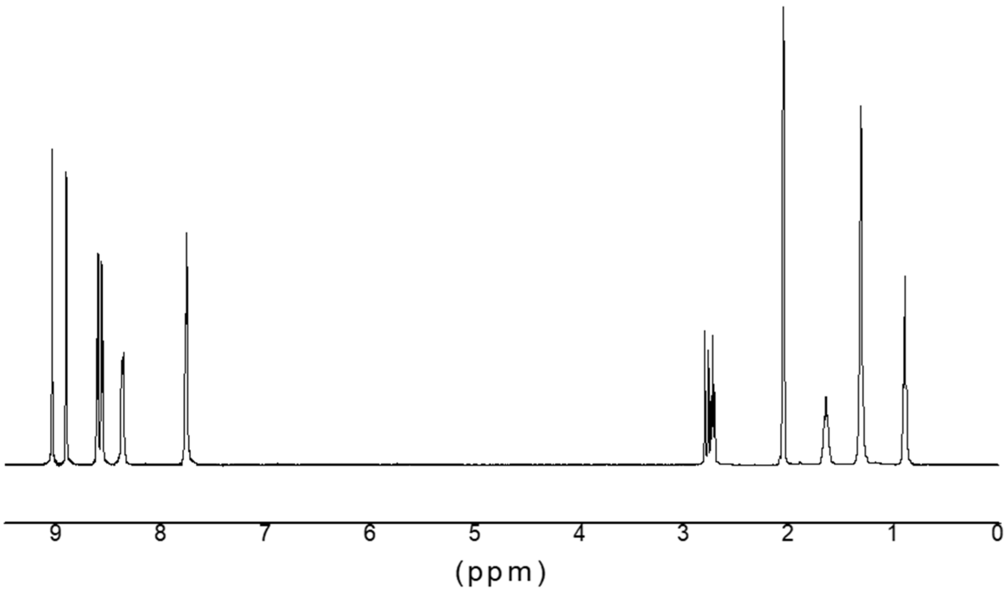

Figure S4. NMR spectrum of compound **4a** in acetone-d$_6$.

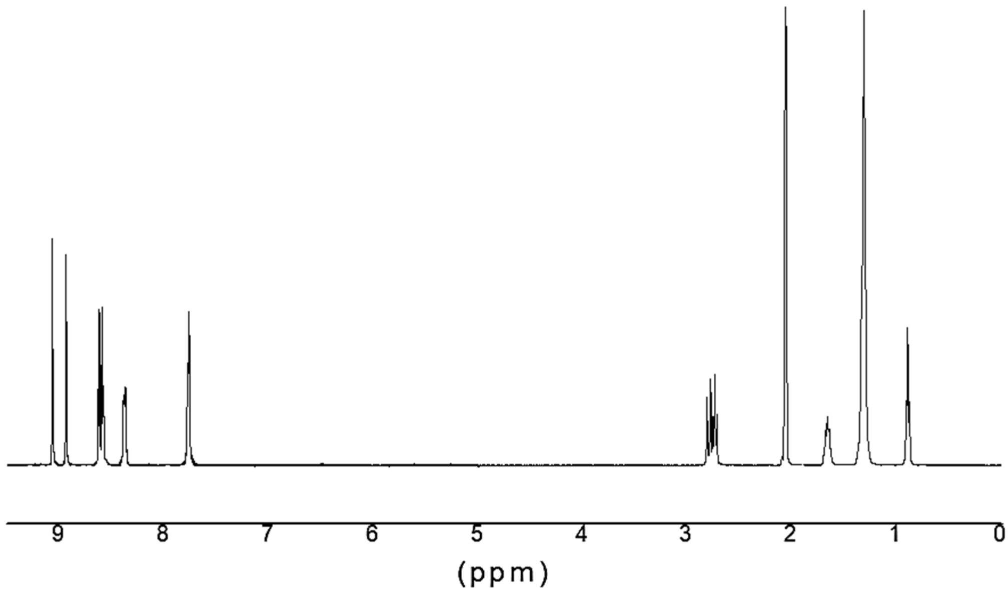

Figure S5. NMR spectrum of compound **4b** in acetone-d$_6$.

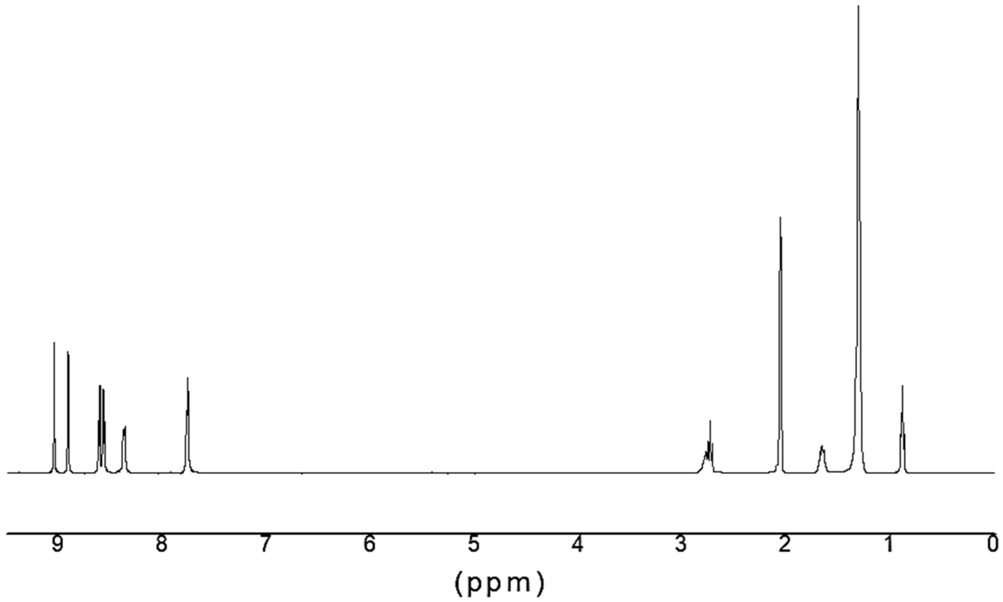

Figure S6. NMR spectrum of compound **4c** in acetone-d_6.

2. Absorption spectra of pyridinium salts 3a-c:

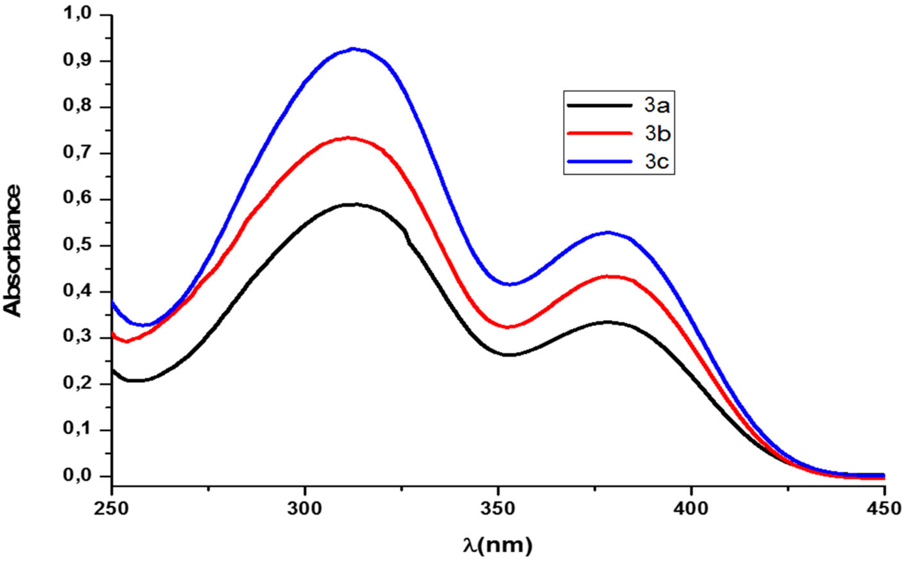

Figure S7. Absorption spectra of pyridinium salts **3a**, **3b** and **3c** in methanol (*ca.* 9 x 10^{-4}M).

3. Cyclic voltammograms of pyridinium salts 3b(a), 3c(b) and BPDTQ salts 4b(a), 4c(b).

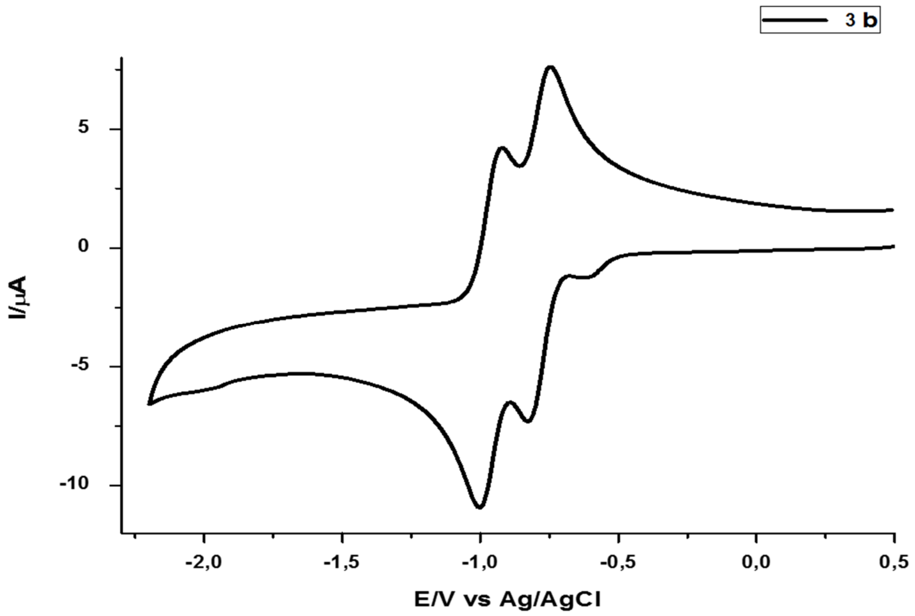

(a)

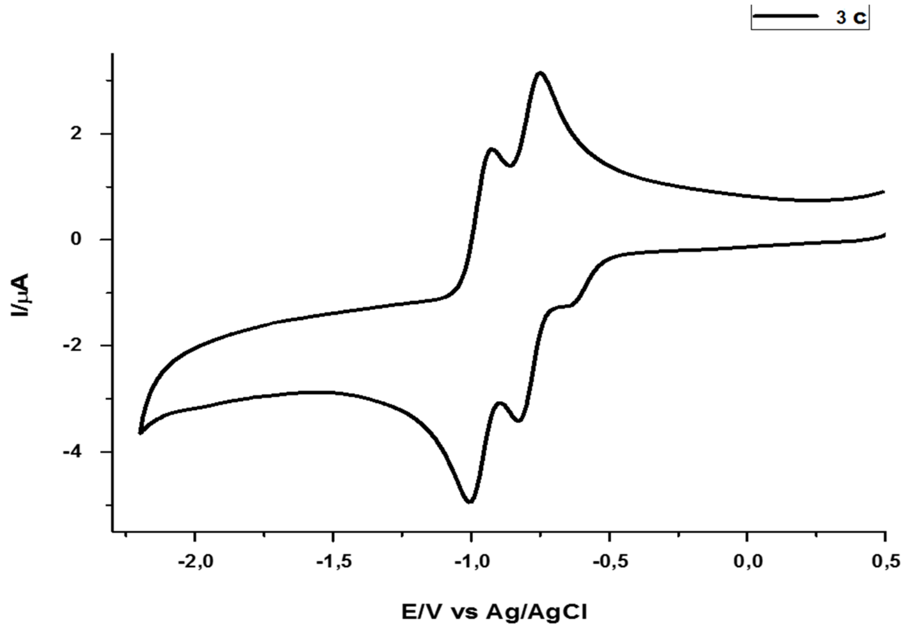

(b)

Figure S8. Cyclic voltammograms obtained for tetrasubtituted pyridinium salts **3b** and **3c** in DMF (c= 1mM), with TBPA 0.1M as supporting electrode and a scan rate of 100mV/s. (**a**) tetrasubtituted pyridinium salts **3b;** (**b**) tetrasubtituted pyridinium salts **3c**.

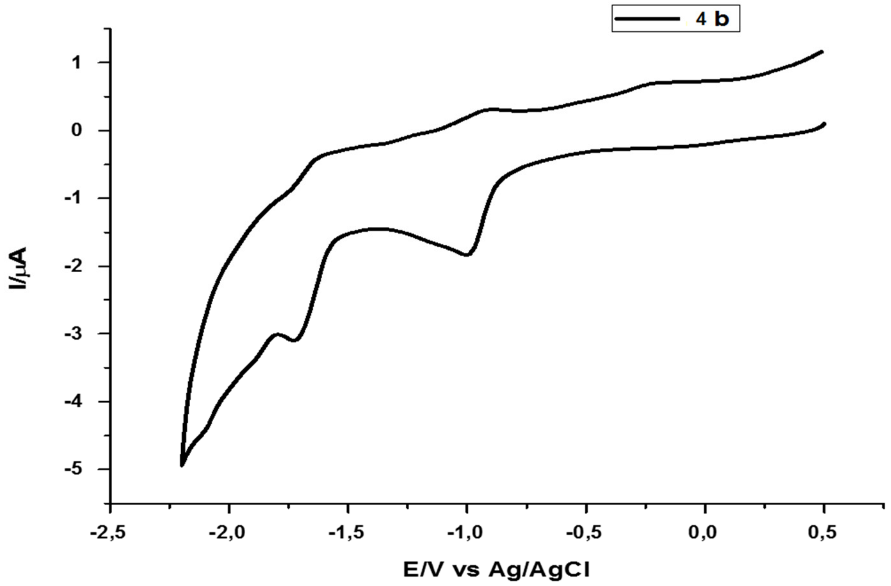

(a)

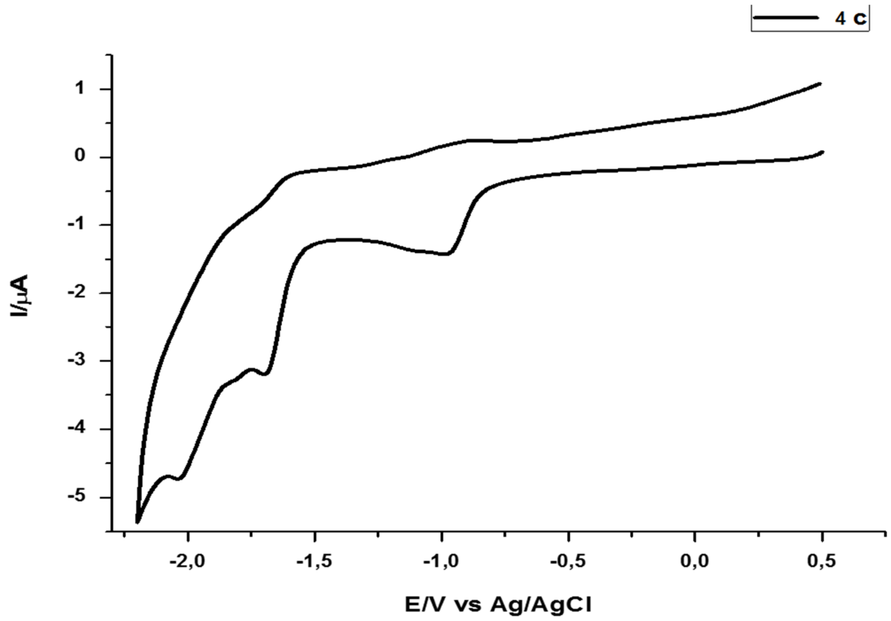

(b)

Figure S9. Cyclic voltammograms obtained for BPDTQ salts **4b** and **4c** in DMF (c= 1mM), with TBPA 0.1M as supporting electrode and a scan rate of 100mV/s. (**a**) BPDTQ salts **4b**; (**b**) BPDTQ salts **4c**.

4. Non covalent interactions plots for face-to-tail (FT) and face-to-face (FF) dimers of 4a-c cations.

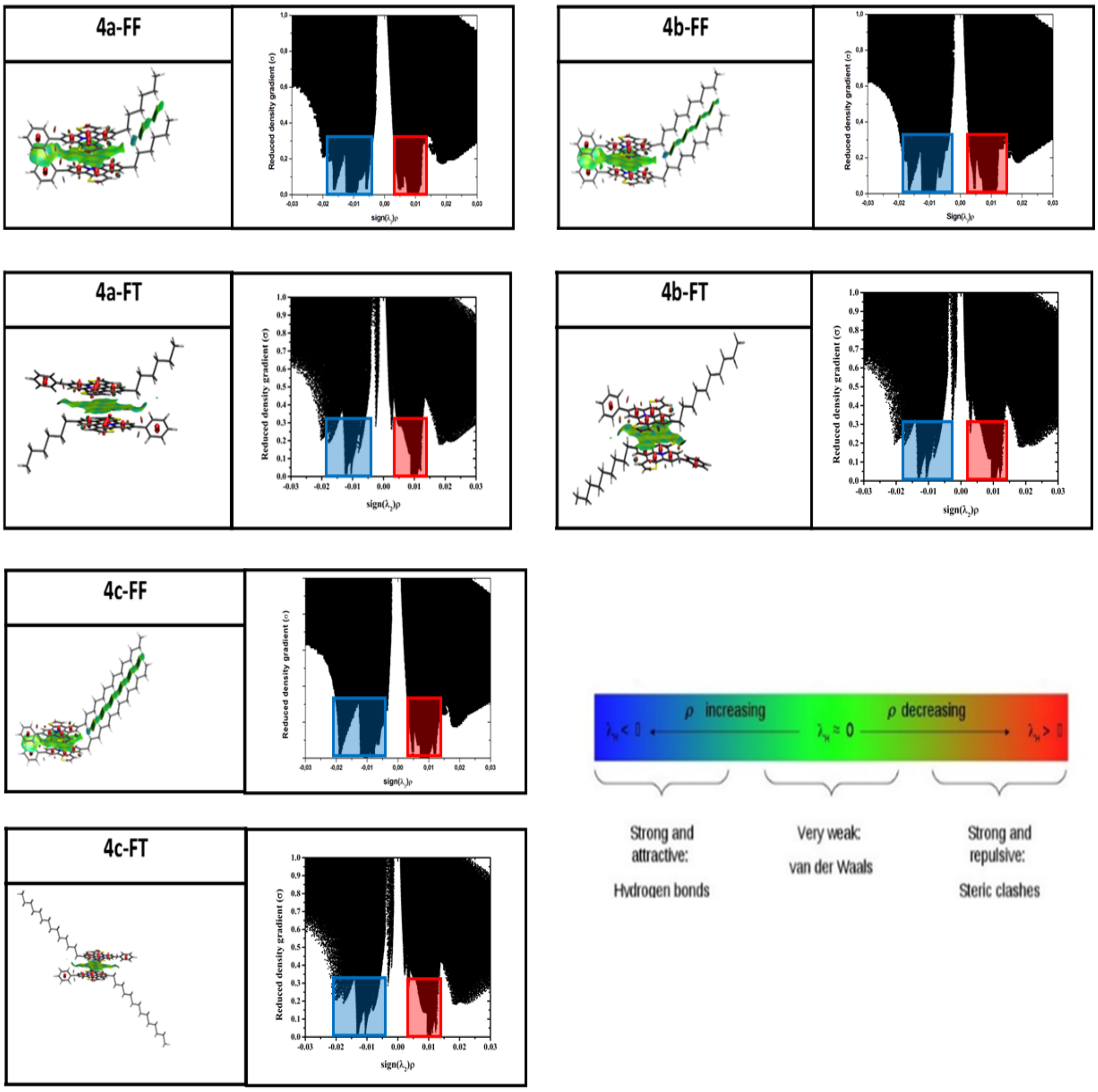

Figure S10. NCI 3D representation isosurfaces and analysis of the $s((\lambda_2)\rho)$ graph for face-to-face (FF) and face-to-tail (FT) dimers. The color-scale code for the 3D representations is shown.

The NCIPlot analysis was done to get a deeper understanding of the stability of the studied $BPDTQ^+$ dimers. In addition, this theoretical analysis is helpful to differentiate the contribution of non-covalent attractive, repulsive, and weak interactions (vdW) within a molecule and in between molecules, which are associated with signals or singularities in the graph of reduced gradient density (s) in the function of the second derivative Hessian eigenvalues $(\lambda_2)\rho$. In this regard, $(\lambda_2)\rho < 0$, $(\lambda_2)\rho \sim 0$ and $(\lambda_2)\rho > 0$, are stated for attractive, weak-interactions, and repulsive interactions, respectively. Additionally, the 3D isosurface representation visually evaluates the nature and localization of the non-covalent interactions following the color scale shown in Figure 10S.

As is observed in Figure 13S, the $s((\lambda_2)\rho)$ graphs at low-density $(\lambda_2)\rho$ and low-reduced gradient (s) values are highlighted zones by red and blue squares. In all cases, vdW and repulsive interactions (π^+-π^+ interactions [1]) are predominant since a considerable signal contribution at low-density and low-reduced gradient at $(\lambda_2)\rho \sim 0$ and $(\lambda_2)\rho > 0$ are observed for the six dimers (red squares in $s((\lambda_2)\rho)$ graphs of Figure 10S). These non-covalent

interactions can also be observed in the 3D isosurface representations showed in Figure 10S for each dimer by the green/light-red disk-like surfaces between the stacked polycyclic pyridinium cores of the dimers. Nevertheless, in each dimer, these interactions are more or less compensated by weak attractive interactions evidenced by the portions of singularities at $(\lambda_2)\rho < 0$. In this regard, the main difference between the face-to-face (FF) and face-to-tail (FT) dimers lies in the non-covalent weak attractive interactions present between the parallel alkyl chains of the FF dimers, with green/light-blue coloured surfaces observed across the entire parallel-chains length. This can be confirmed by the $s((\lambda_2)\rho)$ graphs for the three FF dimers (**4a**, **4b**, and **4c**) that clearly shows a higher signals contribution at low-density and low-reduced gradient in the $(\lambda_2)\rho < 0$ zone (blue squares in $s((\lambda_2)\rho)$ graphs Figure 10S). These additional interactions for the FF dimers, not present in the FT dimers (4a, 4b and 4c), can be associated with their higher electron mobility and, therefore, their possible higher conductivity in their resulting films.

5. Frontier molecular orbitals of BPDTQ+ monomers and dimers

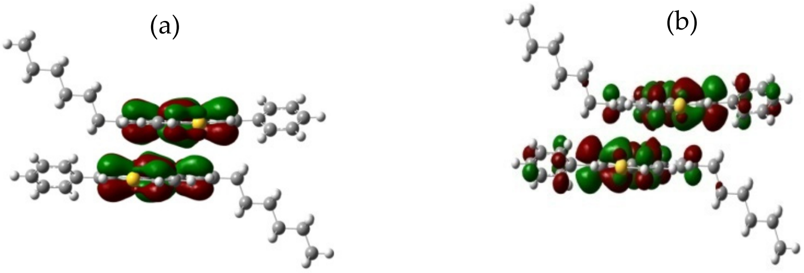

Figure S11. Surfaces of HOMO (**a**) and LUMO (**b**) of FT dimer of **4a+**.

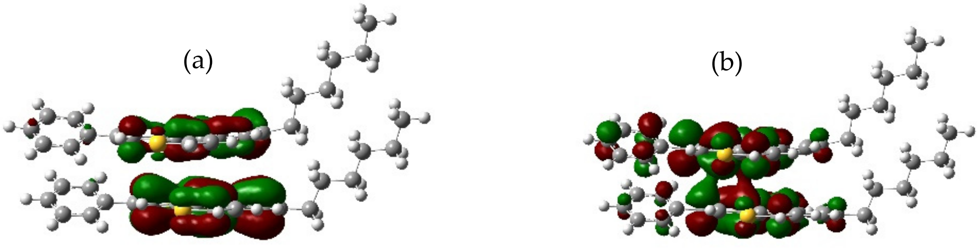

Figure S12. Surfaces of HOMO (**a**) and LUMO (**b**) of FF dimer of **4a+**.

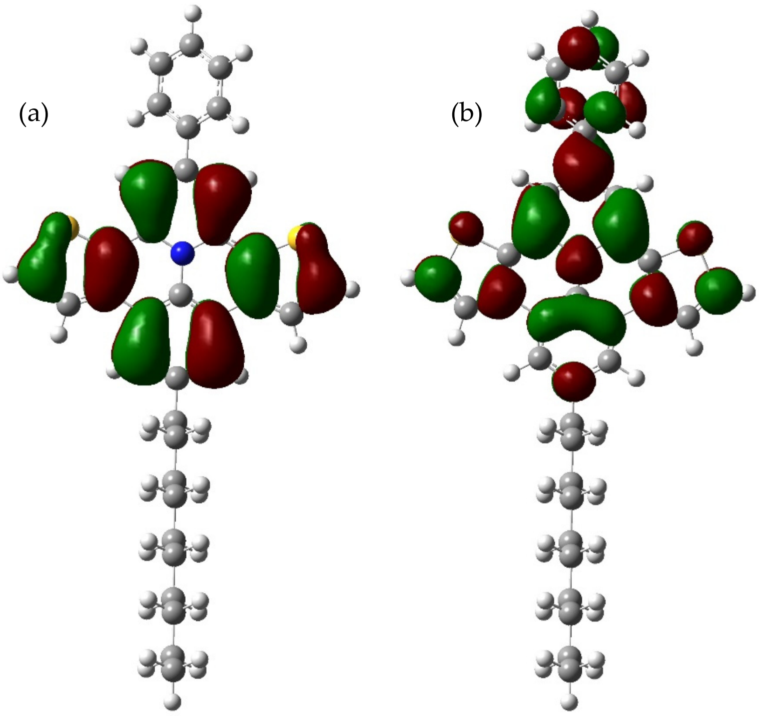

Figure S13. Surfaces of HOMO (**a**) and LUMO (**b**) of **4b**$^+$.

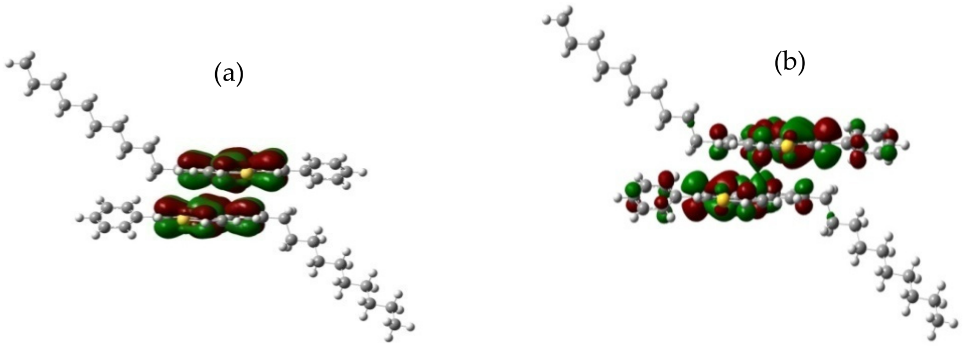

Figure S14. Surfaces of HOMO (**a**) and LUMO (**b**) of FT dimer of **4b**$^+$.

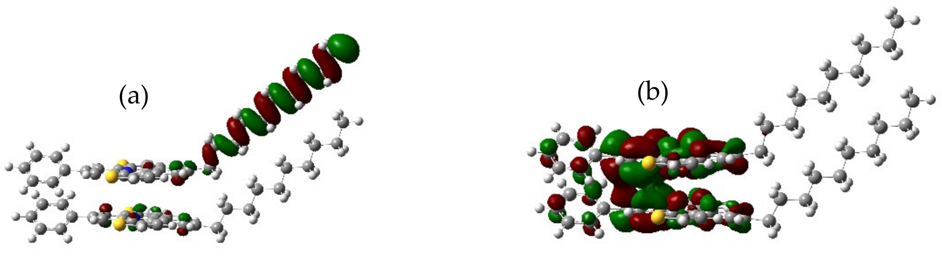

Figure S15. Surfaces of HOMO (**a**) and LUMO (**b**) of FF dimer of **4b**$^+$.

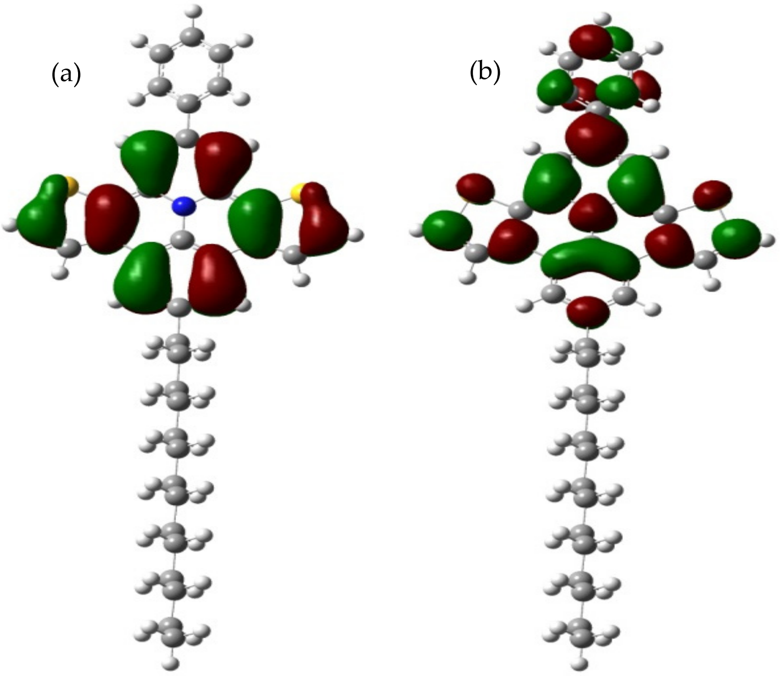

Figure S16. Surfaces of HOMO (**a**) and LUMO (**b**) of **4c⁺**.

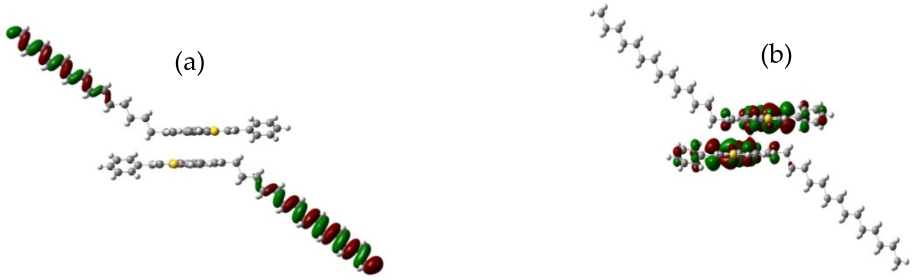

Figure S17. Surfaces of HOMO (**a**) and LUMO (**b**) of FT dimer of **4c⁺**.

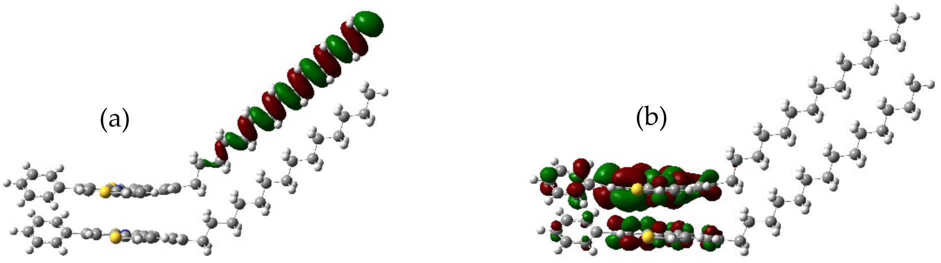

Figure S18. Surfaces of HOMO (**a**) and LUMO (**b**) of FF dimer of **4c⁺**.

Regarding the frontier molecular orbitals for monomeric and dimeric species of **4a-c**, the monomeric BPDTQ cations **4b** (13S) and **4c** (16S) have the same localization of HOMO and LUMO of **4a** (Fig 7), however the distribution of HOMO and LUMO surfaces in dimers FT and FF is very different when the electron-releasing alkyl chain is increased. HOMO surface position in FT dimers **4a** (11S) and **4b** (14S) seems have the same localization of HOMO and LUMO than their respective monomeric species, but when the alkyl chain is increased to C14 in FT dimer **4c** (17S) the HOMO is centered on alkyl chains of each monomer rather different of its monomeric specie HOMO localization and the

LUMO of dimer for each individual monomer continue with the same localization than its monomer **4c** (16S) but the distribution of LUMO in each monomer change slightly as chain length increases from FT dimers **4a-c**. On the other hand, in FF dimer 4a (12S) the HOMO and LUMO surface localization is very similar to the HOMO and LUMO of its monomeric specie **4a** (Fig 7) but the distribution is different in each monomer in both HOMO and LUMO. When the alkyl chain is increased to C10 and C14 in FF dimers **4b** (15S) and **4c** (18S), the HOMO is centered on the alkyl chains of each monomers and the LUMO distribution for each monomer in FF dimers **4b** (15S) and **4c** (18S) also is unequal. This situation shows that when the alkyl chains are increased in the stacked BPDTQ+ species the electron-releasing effect of chains play an important role in the charge transfer process.

References

1. Azizi, A.; Ebrahimi, A. Theoretical investigation of the π + -π + stacking interactions in substituted pyridiniumion. *J. Mol. Graph. Model.* **2017**, *77*, 225–231, doi:10.1016/j.jmgm.2017.08.024.
